# Casein Kinase 2 dependent phosphorylation of eIF4B regulates BACE1 expression in Alzheimer’s disease

**DOI:** 10.1038/s41419-021-04062-3

**Published:** 2021-08-04

**Authors:** Barbara Bettegazzi, Laura Sebastian Monasor, Serena Bellani, Franca Codazzi, Lisa Michelle Restelli, Alessio Vittorio Colombo, Nikolaus Deigendesch, Stephan Frank, Takashi Saito, Takaomi C. Saido, Sven Lammich, Sabina Tahirovic, Fabio Grohovaz, Daniele Zacchetti

**Affiliations:** 1 Vita-Salute San Raffaele University, via Olgettina 58, 20132 Milan, Italy; 2 IRCCS San Raffaele Scientific Institute, via Olgettina 60, 20132 Milan, Italy; 3 German Center for Neurodegenerative Diseases (DZNE) Munich, 81377 Munich, Germany; 4 Basel University Hospital, Institute of Medical Genetics and Pathology, Schoenbeinstrasse 40, 4031 Basel (CH), Switzerland; 5 Laboratory for Proteolytic Neuroscience, RIKEN Center for Brain Science Institute, Wako, Saitama 351-0198 Japan; 6 Department of Neurocognitive Science, Nagoya City University Graduate School of Medical Science, Nagoya, Aichi 467-8601 Japan; 7 BMC – Biochemistry, Ludwig Maximilians University Munich, 81377 Munich, Germany

**Keywords:** Kinases, Alzheimer's disease, Neuronal physiology, Pathogenesis

## Abstract

Alzheimer’s disease (AD) is the most common age-related neurodegenerative disorder. Increased Aβ production plays a fundamental role in the pathogenesis of the disease and BACE1, the protease that triggers the amyloidogenic processing of APP, is a key protein and a pharmacological target in AD. Changes in neuronal activity have been linked to BACE1 expression and Aβ generation, but the underlying mechanisms are still unclear. We provide clear evidence for the role of Casein Kinase 2 in the control of activity-driven BACE1 expression in cultured primary neurons, organotypic brain slices, and murine AD models. More specifically, we demonstrate that neuronal activity promotes Casein Kinase 2 dependent phosphorylation of the translation initiation factor eIF4B and this, in turn, controls BACE1 expression and APP processing. Finally, we show that eIF4B expression and phosphorylation are increased in the brain of APPPS1 and APP-KI mice, as well as in AD patients. Overall, we provide a definition of a mechanism linking brain activity with amyloid production and deposition, opening new perspectives from the therapeutic standpoint.

## Introduction

Alzheimer’s disease (AD), the most common form of dementia, imposes a very high social burden, which has been steadily increasing in the last twenty years. The pathognomonic signs of AD are the accumulation of the amyloid-β peptide (Aβ), with formation of fibrillar extracellular deposits (Aβ plaques) and the intracellular deposition of neurofibrillary tangles, composed by hyperphosphorylated protein tau. According to the amyloid hypothesis [1], extracellular Aβ is the trigger of the pathogenetic process leading to neurodegeneration [2], even though intracellular Aβ might also play a role [3]. The detrimental process started by Aβ is further potentiated by other co-factors, such as Tau accumulation and inflammation [4, 5]. Despite insights provided by biochemical, genetic, and animal model studies, effective therapeutic interventions against AD have not been devised and it is still debated which Aβ species are neurotoxic and which are their cellular targets [6]. However, Aβ-directed therapeutics remain one of the most eagerly sought disease-modifying pharmacological strategies [7–9]. Particular attention has been devoted to BACE1, the enzyme that controls Aβ production by cleaving the amyloid-β precursor protein (APP), and whose dysregulation has been reported in AD patients [10–13]. Despite the promising results of the first attempts of BACE1 inhibition [11, 13], recent studies have pointed out that there are many different BACE1 substrates involved in the regulation of the synaptic function [14], with possible detrimental effects as a consequence of prolonged BACE1 inhibition [15]. Recent data have also shown that the different APP cleavage products are able to influence brain network activity through their interaction with different neuronal receptors [16, 17]. A precise definition of the link between synaptic activity and BACE1 modulation is still needed, even more so after the observation that AD patients treated with BACE1 inhibitors showed a modest worsening of cognitive performance [18–20]. In this respect, the elucidation of the mechanisms controlling BACE1 expression may offer further insights into our understanding of the disease and in the development of therapeutic approaches.

BACE1 is expressed in neurons, especially at the presynaptic terminals [21, 22] and its activity increases with age in mouse, monkey, and non-demented human brain [23]. BACE1 elevation appears to correlate with amyloid pathology and accumulation of BACE1 is observed in normal and dystrophic presynaptic terminals surrounding the amyloid plaques in brains of AD mouse models and patients [21, 22]. Interestingly, neurons around or in contact with amyloid plaques have been reported to show a hyperactive phenotype [24, 25], but evidence of a causative link between synaptic activity and Aβ production is still scarce [26]. Unfortunately, the complexity of the regulation of BACE1 expression in neurons has made it difficult to fully understand mechanisms responsible for BACE1 elevation in AD and to clarify the relationship between Aβ increase and BACE1 upregulation.

Both transcriptional regulation [27] and change in protein stability [28] have been described for BACE1. However, a translational control of its expression seems to be of direct relevance to AD [21, 29]. Reports have highlighted a role for translation initiation [30, 31], with the possible involvement of miRNAs [32–34] and general translation factors, such as eIF2α [35]. This latter possibility, however, was questioned since genetic inhibition of eIF2α phosphorylation was not able to revert the Aβ-dependent BACE1 increase or the amyloid pathology in transgenic AD mice [36]. BACE1 translation largely depends on the presence of its long, conserved, and highly structured transcript leader and is influenced by conditions of cellular stress [37]. Stress stimuli are known to modulate protein synthesis, mostly through the phosphorylation of translation initiation factors, including eIF4B [38]. eIF4B is part of the eIF4 group of translation initiation factors, whose main function is to recruit the mRNA to the ribosome and to assist the scanning of the 43S ribosomal initiation complex. eIF4B has been proposed to favor the interaction of the mRNA with the initiation complex, by binding eIF3 and the 18S rRNA, and to stimulate the eIF4A helicase in the unwinding of secondary structures in the transcript leader of mRNAs [39, 40].

We have recently reported that, in neurons, eIF4B phosphorylation is tuned by the neuronal activity via the control of different kinase pathways. In particular, Casein kinase 2 (CK2)-dependent eIF4B phosphorylation triggers eIF4B localization at synaptic microdomains, increasing eIF4B recruitment to the translation pre-initiation complex and favouring the translation of proteins endowed with highly structured transcript leaders [41]. In this work we demonstrate a causal relationship between neuronal activity, eIF4B phosphorylation and BACE1 expression, and provide evidence for an eIF4B-dependent increase in levels of BACE1 observed in neurons close to Aβ plaques in murine AD models and human brain tissue.

## Results

### Involvement of eIF4B in the regulation of BACE1 expression

There is consensus that the presence of a highly structured and upstream AUG (uAUG) endowed 5′UTR determines an inhibition of BACE1 translation efficiency [30, 42, 43]. However, there is still conflicting evidence about the relative contribution of uAUGs, secondary structures, or the coding sequence in the regulation of BACE1 translatability.

**Fig. 1:**
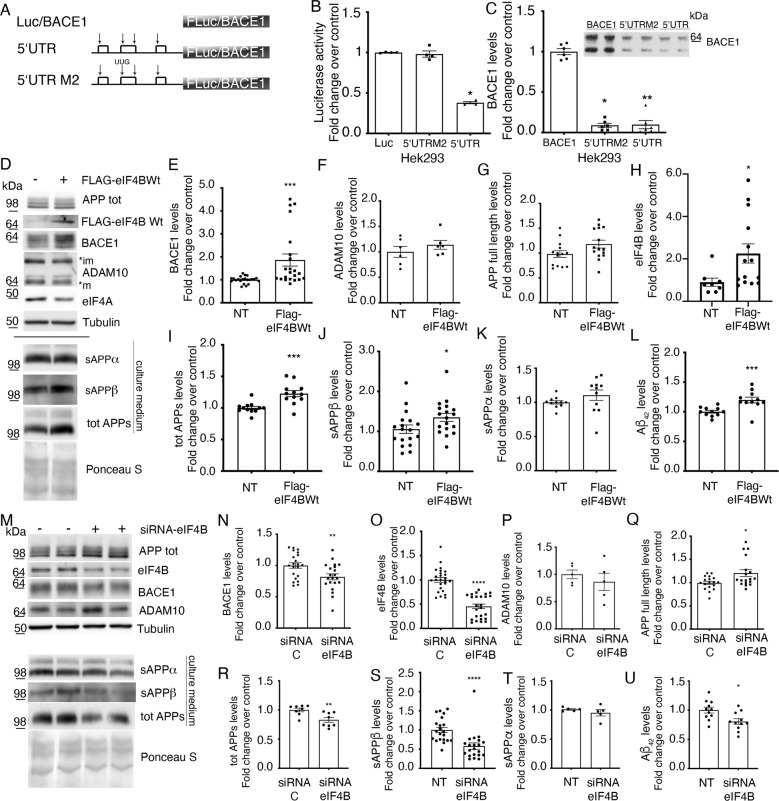
The presence of BACE1 ORF reinforces the translational inhibition of the 5′UTR. **A** Schematic representation of the transfected constructs, containing Firefly luciferase (FLuc) or BACE1 as reporter gene. White boxes represent uORFs and arrows uAUGs. Mutation of the 2nd uAUG to UUG is indicated. **B** Luciferase activity measured in total cell extracts of HEK293 cells, co-transfected with the indicated constructs and a plasmid encoding the renilla luciferase. The Firefly Luciferase activity was normalized on renilla luciferase activity to account for differences in transfection efficiencies and data represent the mean ± SEM of at least three independent experiments, expressed as fold change over control (luciferase). **C** Quantification of BACE1 levels by western blot (inset) in total cell extracts of HEK293 cells, co-transfected with the indicated constructs and a plasmid encoding Firefly luciferase, to normalize for transfection efficiency. Total cell extracts were subjected to luciferase activity measurement and to SDS-PAGE followed by western blot analysis with anti-BACE1 antibody. Blots were then stripped and reincubated with anti-Calnexin antibody as loading control. The intensities of BACE1 band signals were quantified and normalized for loading and for transfection efficiency (on luciferase activity). Results are presented as mean±SEM of at least three independent experiments, with protein levels shown as fold change over control (BACE1). **D**–**T** eIF4B overexpression or silencing modulates the expression of BACE1. Hippocampal neurons were transfected for 96 h either (**D**) with wild-type eIF4B (FLAG-eIF4BWt) or an empty vector (NT) or (**L**) with a control or an eIF4B siRNA pool. **E**–**L**; **N**–**T** Representative western blot and quantification of the indicated proteins are shown. BACE1, ADAM10, eIF4B, full-length APP (lysate), total soluble APP, soluble APP α, or β (culture medium) signals were quantified and normalized for loading (Tubulin for cell extracts, Ponceau S staining for cell culture supernatants). **H** Overexpressed eIF4B levels in transfected neurons were quantified as the sum of endogenous and overexpressed protein. **L**, **U** ELISA measurement of Aβ_42_ levels. Results are presented as mean±SEM of at least three independent experiments, with protein levels shown as fold change over control (cells transfected with empty vector or with control siRNA pool). Statistical significance was calculated using Kruskal–Wallis one-way analysis of variance followed by Dunn’s post hoc test (**B**, **C**), two-tailed Student’s *t*-test (**J**, **N**, **R**, **U**) or Mann–Whitney U test (**E**, **H**, **I**, **L**, **O**, **Q**, **S**) (**B**: *n* = 4 *p* = 0.0145; **C**: *n* = 4 *p* = 0.0145; **E**: *n* = 10 *p* = 0.0003; **H**: *n* = 8 *p* = 0.0113; **I**: *n* = 8 *p* = 0.0005; **J**: *n* = 8 *p* = 0.0485; **L**: *n* = 8 *p* = 0.0006; **N**: *n* = 12 *p* = 0.0082; **O**: *n* = 12 *p* < 0.0001; **Q**: *n* = 12 *p* = 0.0499; **R**: *n* = 8 *p* = 0.0066; **S**: *n* = 8 *p* < 0.0001; **U**: *n* = 8 *p* = 0.0101. *P < 0.05; **P < 0.01; ***P < 0.001; ****P < 0.0001).

The effect of different 5′-UTR sequences (BACE1 native or BACE1 mutated in the 2nd uAUG) on the expression of either luciferase or BACE1 was investigated in both Hek293 cells and primary rat astrocytes (Fig. 1A–C, Fig. S1 A-C).

Panel B of Fig. 1 shows a significant inhibitory effect of BACE1 5′UTR on luciferase activity (∼60%) in Hek293 cells and demonstrates that the effect is prevented by the mutation of the 2nd uAUG. The inhibitory effect of BACE1 5′UTR was even higher (∼90%) in presence of the BACE1 coding sequence and, more notably, it was not influenced by the mutation of the 2nd uAUG (Fig.1C and Fig. S1C).

Based on these results, structural features of the 5′UTR play a more relevant role than uAUGs in BACE1 translation. The eIF4A-dependent helicase activity of the translation initiation complex is crucial for the unwinding of secondary structures in the 5′UTR of mRNAs. Accordingly, we investigated whether the helicase activity of eIF4A could relieve 5′UTR inhibition, focussing our attention on eIF4B, a translation initiation factor that is known to be present in neurons and to stimulate eIF4A [44, 45]. When eIF4B was transfected in primary hippocampal neurons, its overexpression (Fig. 1D, H) was accompanied by a two-fold increase in BACE1, without changes in the levels of the alpha-secretase ADAM10, full-length APP, and other proteins (Fig. 1D–H). In this condition, we detected a slight increase in the soluble form of APP (Fig. 1D–I) that could be attributed to a higher BACE1 activity. Using antibodies able to specifically recognize the different soluble APP peptides, we observed that the levels of the soluble APPβ fragment were increased (Fig. 1D, J), with no change in the levels of soluble APPα (Fig. 1D, K). Interestingly, also Αβ42 levels were increased (Fig. 1L).

Conversely, when endogenous eIF4B levels were reduced by transfecting hippocampal neurons for 96 h with a pool of siRNAs against eIF4B, a reduction in the expression of BACE1 was observed (Fig. 1M–O), accompanied by a slight increase in the levels of the full-length APP (Fig. 1M, Q) and a reduction in the levels of its soluble fragment (Fig. 1M, R). Other proteins, such as AKT or ERK were not significantly affected (Fig. S2). Also in this case we observed that the decrease in sAPP total levels was accompanied by a reduction in soluble APPβ levels and Αβ42 release, again with no change in the levels of soluble APPα (Fig. 1M, S–U).

### Neuronal activity increases BACE1 translation via eIF4B

Multiple BACE1 substrates are localized at the synapse and play an important role in synaptic function [46, 47], supporting the view that BACE1 might be controlled by neuronal activity [48]. Considering the impairment of neuronal connectivity observed in the early phase of AD pathology [49] and the ability of eIF4B to modulate protein synthesis as a function of synaptic activity [41], we investigated whether neuronal activity promotes BACE1 translation via eIF4B.

**Fig. 2:**
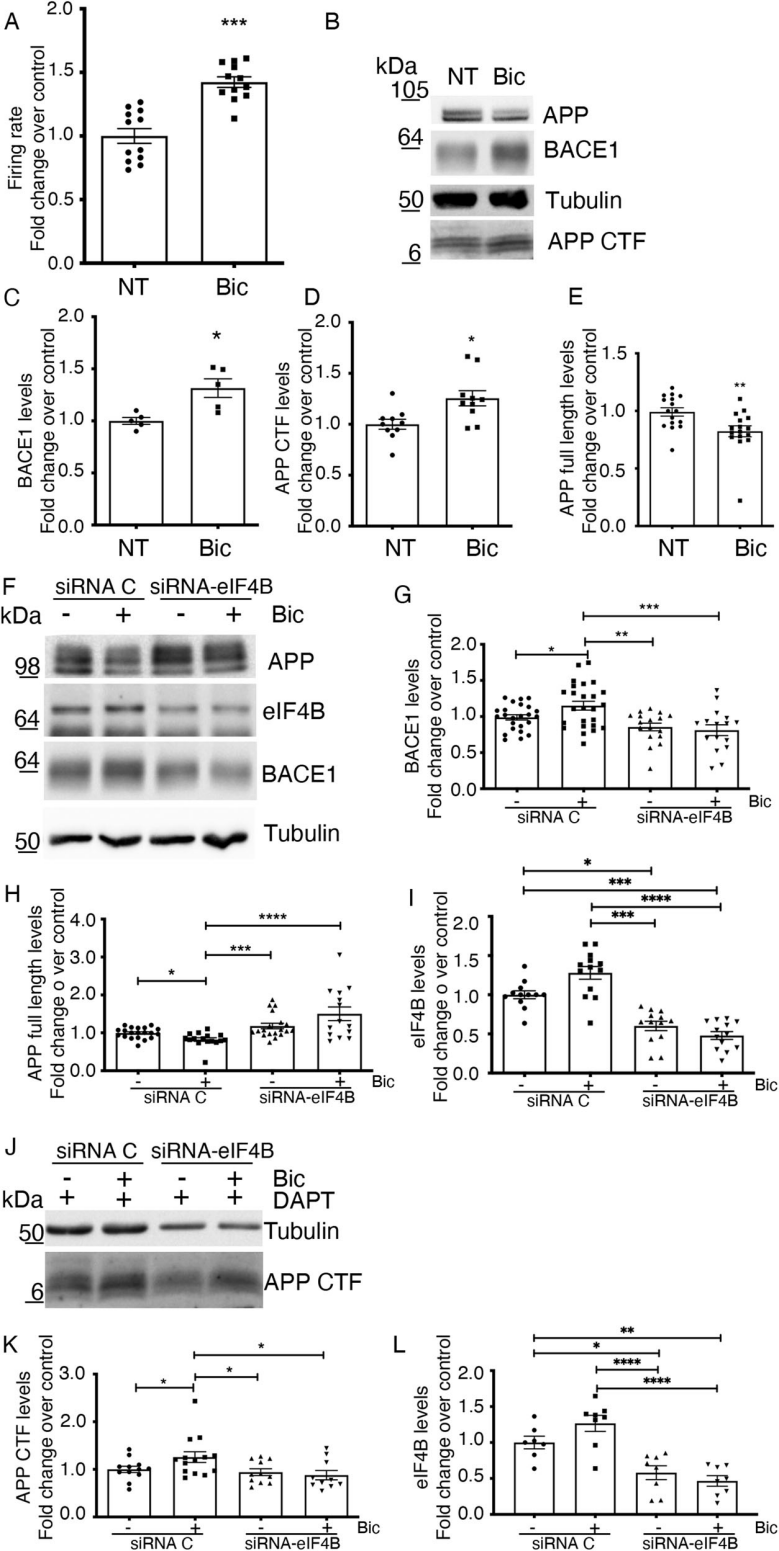
Neuronal activity upregulates BACE1 expression via eIF4B. **A** Average action potential firing rate of neurons treated or not with 10 μM Bic for 20 min as measured by microelectrode array recordings as described in the “Experimental procedure” section; **B**–**E** Representative western blot and quantification of the indicated proteins from total cell extracts of neurons treated or not with 10 μM Bic for 2 h. **F**–**L** Representative western blot and quantification of the indicated proteins from total cell extracts of neurons treated or not with 10 μM Bic for 2 h after 96 h of transfection with the control or eIF4B siRNA pool. **J**–**L** Representative western blot and quantification of the indicated proteins from neurons treated as in (**F**) with the addition of a pretreatment of 30 min with the γ-secretase inhibitor DAPT before administration of Bicuculline. Protein signals were quantified and then normalized for loading (Tubulin). Results are presented as mean ± SEM of at least three independent experiments, with protein levels shown as fold change over control. Statistical significance was calculated by unpaired two-tailed Student’s *t*-test (**A**, **C**, **D**), Mann–Whitney U test (**E**), one-way ANOVA followed by Newman–Keuls post hoc test (**G**, **K**), and Kruskal–Wallis one-way analysis of variance followed by Dunn’s post hoc test (**H**, **I**, **L**) (**A**: *n* = 12 *p* = 0.0001; **C**: *n* = 5 *p* = 0.0111; **D**: *n* = 10 *p* = 0.0105; **E**: *n*= 16 *p* = 0.0029; **G**: *n* = 12 *p* = 0.0002; **H**: *n* = 9 *p* < 0.0001; **I**: *n*=9 *p*<0.0001, **K**: *n* = 6 *p* = 0.0244; **L**: *n* = 6 *p* <0.0001; *P < 0.05; **P < 0.01; ***P < 0.001; ****P < 0.0001).

Treatment of the hippocampal neurons with 10 μM bicuculline (Bic), an antagonist of the GABA_A_ receptor, increased both excitatory and intracellular calcium activities, as evaluated by microelectrode arrays (Fig. 2A), as well as BACE1 levels (Fig. 2B, C). Increased neuronal activity promoted an increase in the levels of carboxyl-terminal fragments of APP (APP-CTF), measured under conditions of γ-secretase inhibition with DAPT (see the “Experimental procedure” section), with a concomitant slight reduction in the levels of full-length APP (Fig. 2B–E).

The increase in the levels of BACE1 was prevented by pretreatment with the pool of siRNAs against eIF4B (Fig. 2F, G). The knockdown of eIF4B (Fig. 2F–I, J–L) was also able to prevent the reduction in full-length APP levels promoted by bicuculline treatment (Fig. 2F, H) and the increase in APP-CTF levels (Fig. 2J, K).

Taken together, these results suggest a cascade of events in which synaptic activity promotes eIF4B activation, with ensuing increase in BACE1 expression and APP processing, possibly up to Aβ production.

### Casein Kinase 2 mediates eIF4B-induced BACE1 upregulation

**Fig. 3:**
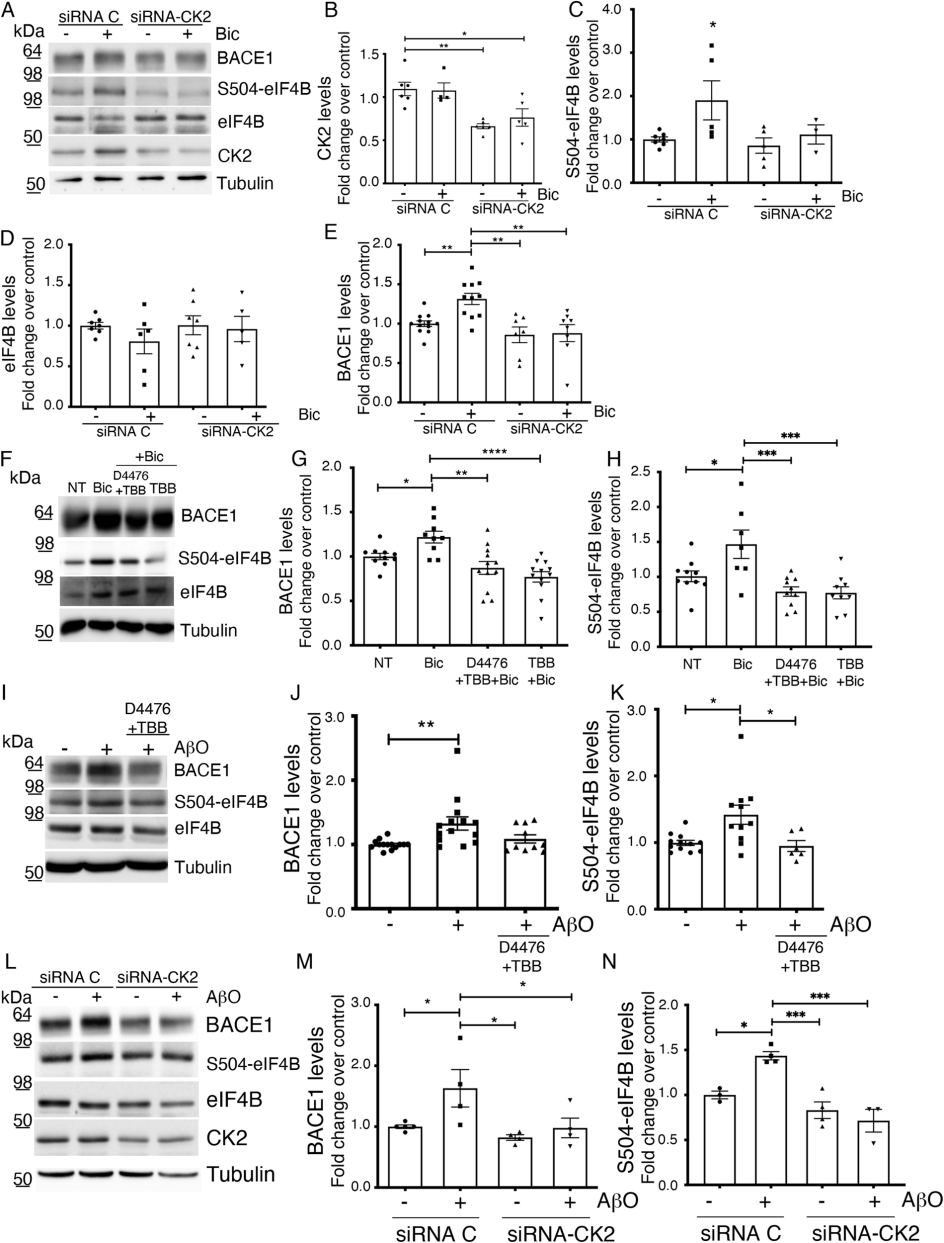
CK2 silencing or inhibition downregulates BACE1 expression induced by neuronal activity or Aβ challenge. Representative western blot and quantification of the indicated proteins in extracts from primary hippocampal neurons: **A**–**E** treated or not with 10 μM Bic for 2 h, without or with pretreatment with the CK2 siRNA pool for 96 h; **F**, **G** pre-treated or not for 4 h with a CK2 inhibitor (25 μM TBB) alone or in combination with a CK1 inhibitor (10 μM D4476, 25 μM TBB) and analyzed after treatment with 10 μM bicuculline (Bic) for 2 h; **I**–**K** pre-treated or not for 4 h with a CK2 inhibitor (25 μM TBB) alone or in combination with a CK1 inhibitor (10 μM D4476, 25 μM TBB) and analyzed after treatment with 2.5 μM Oligomeric Aβ (AβO) for 2 h; **L**–**N** treated or not with 2.5 μM Oligomeric Aβ (AβO) for 2 h, without or with pretreatment with the CK2 siRNA pool for 96 h. Protein levels were quantified, normalized for loading (Tubulin), and then shown as fold change over control (untreated neurons). Phosphorylated eIF4B levels are normalized against total eIF4B, then for loading (Tubulin) and shown as fold change over control (untreated neurons). Data are shown as mean ± SEM of at least three independent experiments for each treatment. Statistical significance was calculated by one-way ANOVA followed by Tukey’s (**B**), Dunnett (**C**, **H**, **N**), Newman–Keuls post hoc test (**G**, **M**), nonparametric Kruskal–Wallis one-way analysis of variance followed by Dunn’s post hoc test (**E**, **J**, **K**) (**B**: *n* = 4 *p* = 0.0007; **C**: *n* = 4 *p* = 0.00410; **E**: *n* = 4 *p* = 0.0013; **G**: *n* = 5 *p* < 0.0001; **H**: *n* = 5 *p* = 0.0004; **J**: *n* = 5 *p* = 0.0071; **K**: *n* = 5 *p* = 0.0096; **M**: *n* = 4 *p* = 0.0288; **N**: *n* = 4 *p* = 0.0004; *P < 0.05, **P < 0.01, ***P < 0.001).

In a previous work, we demonstrated that Casein Kinases mediate the neuronal-specific phosphorylation of eIF4B on the Ser504 residue and that this phosphorylation increases the recruitment of eIF4B to the translation initiation complex [41]. Based on this evidence, we tested whether silencing or pharmacological inhibition of Casein Kinase 2 was able to interfere with the eIF4B-induced BACE1 upregulation. As we predicted, knockdown of CK2 with specific siRNA for 96 h in primary hippocampal neurons prevented the activity-induced increase in eIF4B Ser504 phosphorylation, without affecting total eIF4B levels, as well as the increase in BACE1 expression induced by bicuculline treatment (Fig. 3A–E). As expected from their reciprocal regulation [50], CK2 downregulation produced a reduction of AKT phosphorylation on Ser473, leaving unaffected the levels of phosphorylated ERK (Fig. S3). Similarly, pharmacological inhibition of CK2 (25 μM TBB) or CK1 + CK2 (10 μM D4476 + 25 μM TBB), a condition that is even more effective in inhibiting eIF4B phosphorylation (Fig. 3F–H), also prevented BACE1 upregulation upon Bic treatment (Fig. 3F, G).

Neurons around Aβ plaques are reported to be in a hyperactive state [24, 25] and to express high levels of BACE1 [22, 51]. In addition, soluble Aβ_42_ has been shown to promote neuronal hyperexcitability [52, 53], while soluble Aβ oligomers increased BACE1 levels [54, 55]. To evaluate a possible role of eIF4B in the Aβ mediated BACE1 upregulation, we treated mature hippocampal neurons with oligomeric Aβ (2.5 μM for 2 h). In this respect, our data confirmed previous reports, with neurons exposed to soluble oligomeric Aβ species showing an upregulation of BACE1 (Fig. 3I, J). More interestingly, these neurons also showed higher levels of eIF4B phosphorylation at the Ser504 site (Fig. 3I, K). Silencing (with siRNAs) or pharmacological inhibition of Casein Kinase before oligomeric Aβ stimulation prevented the increase in both Ser504 phosphorylation (Fig. 3I, K, L, N) and BACE1 levels (Fig. 3I, J, L, M) providing further evidence of the relevance of this enzyme in the control of BACE1 levels through eIF4B phosphorylation.

### Upregulation of eIF4B phosphorylation in AD mouse models

**Fig. 4:**
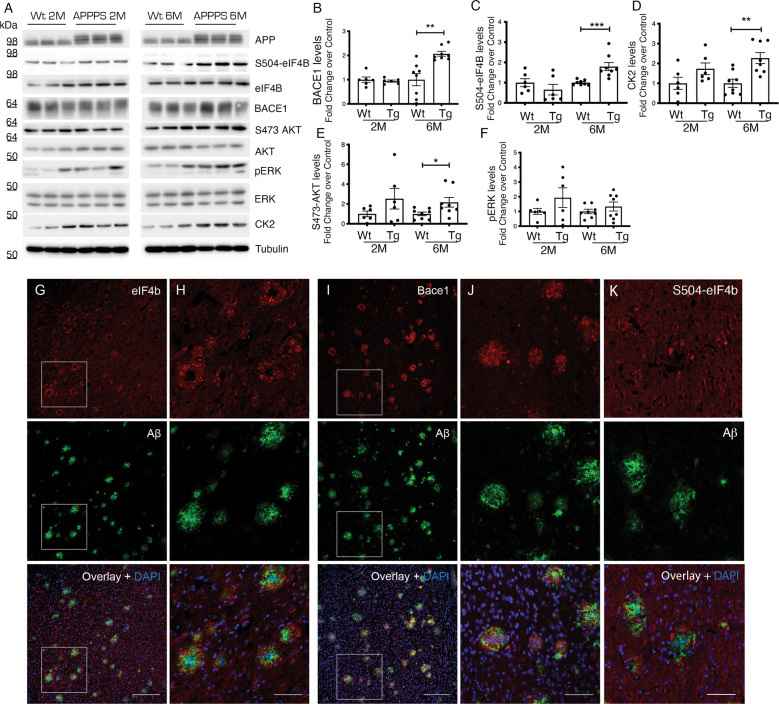
Increased eIF4B expression and phosphorylation in the brains of APPPS1 mice. **A**–**F** Representative western blot (**A**) and quantification (**B**–**F**) of the indicated proteins in extracts from 2 (2 M) or 6 (6 M) months old brains from APPPS1 (APPPS) or wild-type (Wt) mice. Protein levels are shown as fold change over control (Wt mice). Levels of phosphorylated proteins are normalized against the corresponding total protein, then for loading (Tubulin) and shown as fold change over control (Wt mice). Statistical significance was evaluated by unpaired two-tailed Student’s *t*-test. **G**–**J** Immunohistochemical analysis of eIF4B expression in 6-month-old APPPS1 cortices immunostained for eIF4B (red: **G**, **H**) or BACE1 (red: **I**, **J**) or S504-eIF4B (**K**) in combination with Aβ (green) revealed a localization of eIF4B in close proximity of plaques, with a pattern similar to the distribution of BACE1 in dystrophic neurites. Second and fourth column panels are higher magnification images of boxed regions. Scale bars: **G**, **I**, 50 μm, **H**, **J, K** 150 μm (2 months *n* = 6 mice for each genotype; 6 months *n* = 8 mice for each genotype; **B**: *p* = 0.0019; **C**: *p* = 0.0003; **D**: *p* = 0.0047; **E**: *p* = 0.0207; **P < 0.01, ***P < 0.001, ****P < 0.0001).

The role of eIF4B in the regulation of BACE1 expression was further investigated in vivo, using two commonly explored AD mouse models. We evaluated eIF4B and BACE1 levels by western blot and immunohistochemistry in brains of APPPS1 mice [56] at two time points: 2 months, representative of a stage with scarce pathology and 6 months, representing fully established AD pathology. Similarly, as demonstrated in vitro, our in vivo analysis also shows a clear correlation between the increase in eIF4B phosphorylation and BACE1 expression (Fig. 4A–C). At 6 months, eIF4B phosphorylation was elevated at Ser504 site (Fig. 4A, C) and was paralleled by an increase in the levels of CK2 and AKT phosphorylation in Ser473 (Fig. 4D–F).

We then moved to analyze eIF4B and BACE1 localization around Aβ plaques on contiguous sections of APPPS1 mice brains. Interestingly, eIF4B and BACE1 expression could be detected around Aβ plaques (Fig. 4G, H), with a similar pattern (Fig. 4I, J). The same kind of immunolocalization was observed in neurons labeled with the antibody against S504-eIF4B (Fig. 4K).

We further validated this result on brains of 2 and 6 months old *App*^*NL-G-F*^ mice, a knock-in animal model characterized by endogenous levels of APP and a humanized Aβ sequence where human AD mutations have been introduced [57]. In this AD model at 6 months, an increase in eIF4B phosphorylation was accompanied by an elevation in BACE1 expression levels (Fig. S4A-C). The levels of CK2 were also increased at the 6 months time point (Fig. S4A, D, F), with no changes in the levels of S473 phosphorylated AKT or pERK (Fig. S4A, E, F). Also in the *App*^*NL-G-F*^, the pattern of eIF4B localization around plaques closely resembled the distribution of BACE1 around Aβ plaques (Fig. S5A-D), with some dystrophic neurites in the plaque vicinity displaying also higher levels of Ser504-eIF4B phosphorylation (Fig. S6).

Altogether, our analysis of AD models strengthens a role for eIF4B in the regulation of BACE1 expression that is known to be found increased in the dystrophic neurites surrounding the Aβ plaques.

### Treatment of organotypic brain slice cultures from APPPS1 mice with CK inhibitors reduces BACE1 levels

**Fig. 5:**
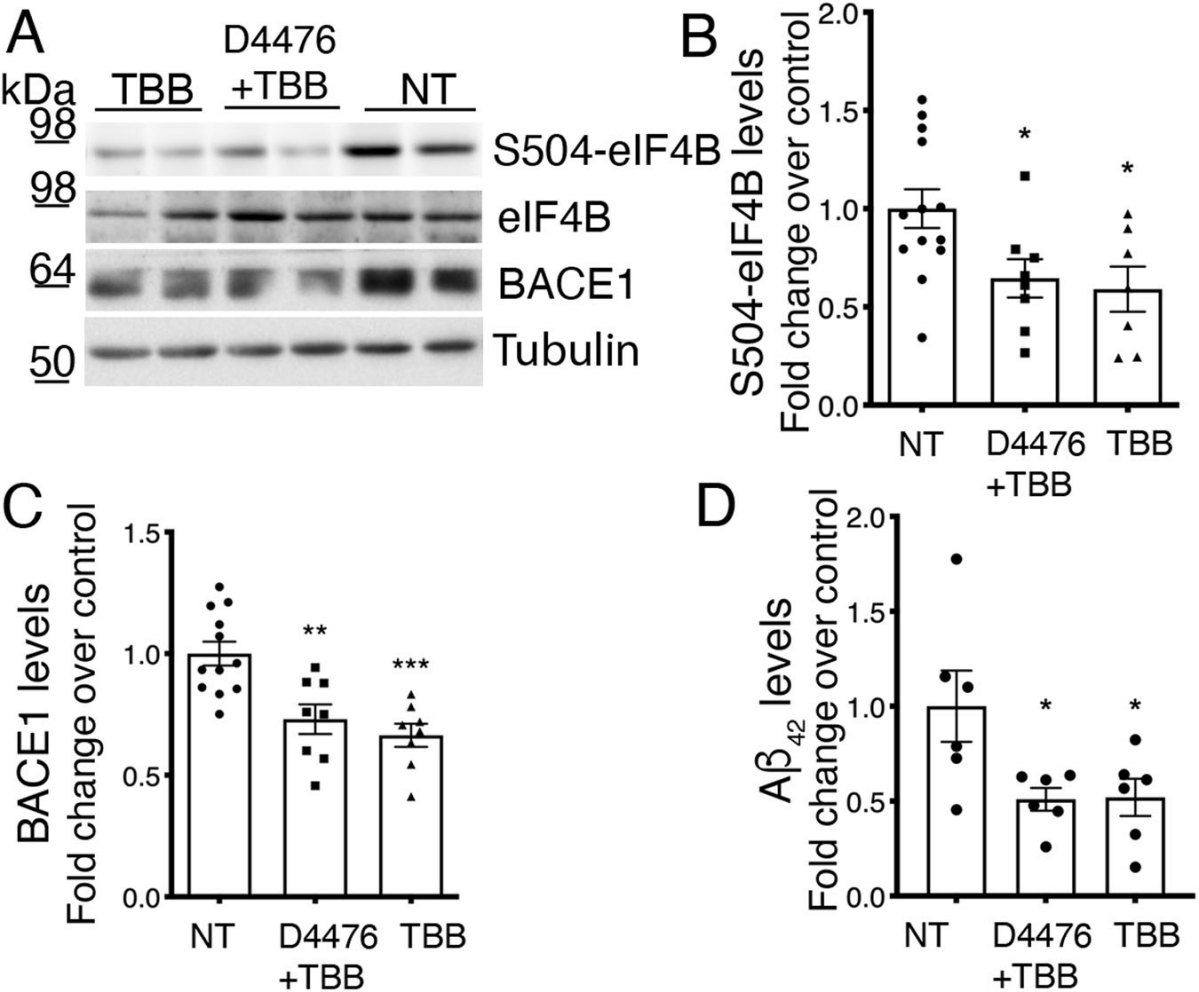
Casein Kinase inhibition lowers BACE1 levels in organotypic slices from APPPS1 mice. **A**–**C** Western blot analysis for eIF4B total or phosphorylated (S504), BACE1, and Tubulin performed on extracts from APPPS1 brain slices treated with CK2 inhibitor (TBB), alone or in combination with CK1 inhibitor (D4476+TBB) or left untreated (NT). Protein levels are shown as fold change over control (untreated slices). Levels of phosphorylated proteins are normalized against the corresponding total protein, then for loading (Tubulin) and shown as fold change over control (untreated slices). **D** ELISA measurement of Aβ_42_ levels in culture supernatants of slices treated as in (**A**). Slices were treated for 7 days before analysis. Statistical significance was evaluated by one-way ANOVA followed by Dunnett post hoc test (**B**: *n* = 6 *p* = 0.0161; **C**: *n* = 6 *p* = 0.0002) or Tukey post hoc test (**D**: *n* = 6 *p* = 0.0235).

Organotypic brain slices from an AD mouse model were reported to develop some AD-like features in culture [58]. Although it is known that culturing of neonatal organotypic brain slices from AD mice does not recapitulate Aβ plaque deposition, likely due to efficient Aβ clearance [59–61], some of the AD-like features can be studied using this ex vivo model. Therefore, we decided to evaluate the effect of CK inhibition in this type of experimental model, which is more representative of the physiopathological condition. The effect of CK inhibitors (10 μM D4476 + 25 μM TBB; 1-week treatment) was evaluated on mouse organotypic cortico-hippocampal brain slices obtained from P8 APPPS1 mice cultured for 18–20 days. To exclude the possibility that this prolonged treatment had toxic effects on the organotypic cultures, we measured lactate dehydrogenase (LDH) release into the culture medium: neither D4476 nor TBB significantly affected total LDH release (Supplementary Fig. S7), indicating that CK inhibitors do not cause toxicity in cultured brain slices at these concentrations. The treatment with the CK inhibitors significantly reduced the levels of eIF4B phosphorylation on Ser504 (Fig. 5A, B) as well as the levels of BACE1 protein (Fig. 5A, C) and Aβ_42_ levels (Fig. 5D), confirming the data obtained in primary neuronal cultures and in the APPPS1 mice brains.

### Upregulation of eIF4B expression and phosphorylation in human AD brains

**Fig. 6:**
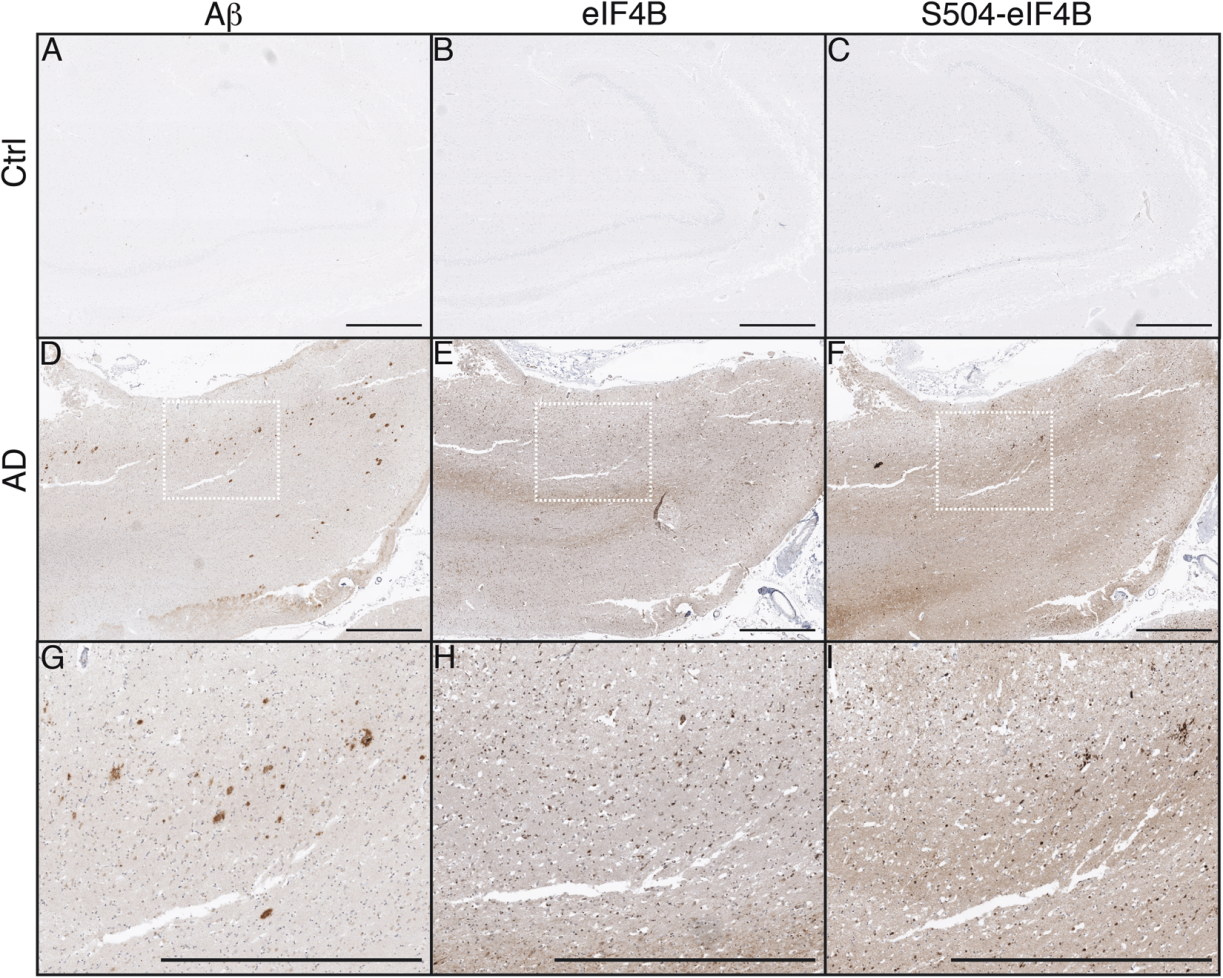
Expression and phosphorylation of eIF4B are increased in human AD hippocampi. Representative immunohistochemical analysis of human AD and control hippocampal sections (*n* = 3) stained for Aβ (**A**, **D**, **G**), total (**B**, **E**, **H**), and phospho Ser504 eIF4B (**C**, **F**, **I**) showing that both total and phosphorylated eIF4B are increased in the presence of Aβ pathology. **G**–**I** panels are higher magnification images of boxed regions. Scale bars: 1 mm.

To further explore the relevance of eIF4B-dependent BACE1 upregulation in AD patients, we performed an experiment on formalin-fixed, paraffin-embedded tissues obtained from human AD and control hippocampi (*n* = 3). Figure 6 shows representative sections that confirm that both total and Ser504-phosphorylated eIF4B are increased in the presence of Aβ pathology. Furthermore, the non-phosphorylated protein staining appears stronger in the pyramidal layer while phospho-eIF4B signal appears more enriched in the dendritic regions (Fig. 6G–I).

## Discussion

BACE1 translation is known to be regulated [10, 62]. It has been shown that the 5′UTR of BACE1 harbours an intrinsic repressing activity of translation, which was initially ascribed to the contribution of uAUGs and upstream open reading frames (uORFs) combined with the structural hindrance given by the high GC-content [30, 42, 43]. Our data confirm the inhibitory role of the 5′UTR on BACE1 translation, showing however that BACE1 coding sequence or, at least, the region at the 5′UTR-ORF border, has relevant inhibitory properties that are potentially related to structural elements. This hypothesis is substantiated by the absence of a translational rescue operated by the second uAUG mutation, when BACE1 coding sequence was used as a reporter. In fact, in Hek293 cells, the BACE1 coding sequence appears to introduce an additional inhibitory element that cannot be overcome by the mutation of the second uAUG. This additional inhibitory effect could be due to the formation of an even more complex structured region, as suggested by in silico secondary structure prediction, or the binding of specific cellular factors; both possibilities are worthy of further investigation.

BACE1 translational repression, which is constitutively active, is expected to be relieved under specific conditions. A likely possibility is that stimuli able to modulate BACE1 translation may influence the helicase activity of the eIF4F complex, thus allowing a better unwinding of the structured 5′UTR and a more efficient scanning by the small ribosomal subunit. For this reason, we focused our attention on eIF4B, whose role in the regulation of translation of structured transcripts has recently gained attention [40, 63]. eIF4B function in the translation process is influenced by phosphorylation [64, 65] and we have recently shown that increase in synaptic activity changes the phosphorylation state of eIF4B, in a way that both its interaction with the translation initiation complex and its localization at the synaptic sites are favored [41].

Considering that not only eIF4B mRNA and protein [66, 67], but also BACE1 protein [22] are present at synapses, we investigated whether, upon neuronal activation, eIF4B phosphorylation was able to boost the translation of BACE1.

Our results demonstrate that a moderate increase in spontaneous neuronal activity, obtained by antagonizing the inhibitory connections of an in vitro neuronal network, triggers the phosphorylation of eIF4B and, in turn, enhances BACE1 expression, promoting APP processing. Interestingly, only the amyloidogenic pathway appeared to be potentiated while neither APP levels nor non-amyloidogenic processing were affected. All these findings draw the attention to the influence exerted by the neuronal activity on BACE1 expression and its synaptic localization.

Neuronal activity is considered a trigger for the amyloidogenic pathway [68–70] and it was shown to promote the interaction between APP and BACE1 [71]. A correlation between altered brain activity and amyloid deposition has already been shown in patients [72, 73] and alterations in the activity of specific brain networks, including epileptiform discharges [74], were observed during the early phase of AD [73]. Altogether, a control of BACE1 expression in the synaptic context might offer the possibility to intervene on Aβ deposition without influencing the physiological events downstream of the APP processing pathway.

Starting from this complex scenario, here we show that both an increase in neuronal network activity and administration of soluble forms of Aβ promote eIF4B phosphorylation, BACE1 expression and APP processing. The physiological relevance of a proper control of this pathway is further supported by the evidence that eIF4B phosphorylation is increased in the brain of APPPS1 and APP-KI mice. Even more striking is the fact that this occurs also in the brain of AD patients. More data will be necessary to strengthen this finding and to determine whether the increase in phospho-eIF4B is the main cause of BACE1 elevation in AD mice.

This body of evidence fits with a general scenario in which increased soluble Aβ levels and/or subtle alterations of the neuronal activity sustain CK2 activation and eIF4B phosphorylation at the synapse, possibly in an early phase of the disease, with ensuing increase in BACE1 expression and Aβ production. With disease progression, the accumulation of eIF4B and BACE1 around plaques is expected to constitute the premise of a vicious circle of increased Aβ production followed by eIF4B hyper-phosphorylation and BACE1 elevation. In this respect, it has recently been shown that Αβ_42_ is able to promote changes in axonal protein translation [75] and that local protein synthesis at synapses was found altered in a mouse model of AD [76].

Our hypothesis of a eIF4B-dependent regulation of BACE1 expression is also strengthened by the recent observation that eIF4B phosphorylation plays a role in the modulation of BACE1 levels promoted by matrix metalloproteinase 13 [77].

Since BACE1 transport and subcellular localization are also a critical step in amyloidogenesis [78, 79], we cannot exclude that eIF4B and BACE1 accumulation in proximity of plaques could depend on mechanisms other than local translational regulation, such as an alteration of the axonal transport, an early sign of AD pathology [80].

BACE1 inhibition was considered the most promising strategy for lowering Aβ burden in the brain of AD patients, but the adverse effects that led to the discontinuation of several clinical trials fell short of everybody’s expectations [18–20]. However, in those studies, BACE1 activity was inhibited up to 90% in patients with already established Aβ deposition.

It remains to be established if a partial reduction of BACE1 levels, possibly before the appearance of amyloid pathology, may prevent these detrimental side effects, maintaining the cleavage of substrates other than Aβ and preserving the physiological functions of the enzyme.

Our findings provide a novel view on how BACE1 expression is regulated by synaptic activity, identifying CK2 and eIF4B as key players. The possibility to use CK2 inhibitors to specifically control BACE1 increase, upon synaptic activation or presence of excess Aβ, may thus represent a therapeutic target different from those currently assessed in AD and with a disease-modifying potential. Testing CK2 inhibition in vivo in a mouse model of AD will be a necessary step to take: CK2 is a pleiotropic kinase and we are aware that its inhibition poses other relevant questions on possible side effects. However, the fact that specific CK2 inhibitors are currently used in phase I and II clinical trials for cancer therapy, may significantly accelerate the possibility to test the potential of these drugs to prevent and/or treat AD in its early phase. Last, the possibility to use gene therapy protocols (i.e., nasal delivery of viral particles) or specific delivery systems (conjugation with nanoparticles) aimed at delivering shRNAs against CK2 or eIF4B in a cell specific and temporally restricted way could open new interesting possibilities to selectively target this pathogenetic processes in AD.

## Experimental procedure

References for the “Experimental procedure” section are reported in the Supplementary material file.

### Animals

Hemizygous APPPS1 mouse line overexpressing human APPKM670/671NL and PS1L166P under the control of the Thy-1 promoter [1], homozygous *App*^*NL-G-F*^ mouse line [2], and the corresponding C57BL/6J (WT) line were used in this study. Mice were group-housed under specific pathogen-free conditions. Mice had access to water and standard mouse chow (Ssniff® Ms-H, Ssniff Spezialdiäten GmbH, Soest, Germany) ad libitum and were kept under a 12/12 h light-dark cycle in IVC System. All animal experiments were performed in compliance with the German animal welfare law and have been approved by the government of Upper Bavaria. All animal experiments have been carried out following the ARRIVE guidelines and in accordance with the EU Directive 2010/63/EU for animal experiments. For the 2 months time point 6 animals per group were used (Wt, APPPS1, and *App*^*NL-G-F*^), 4 females and 2 males for each genotype. For the 6 months time point 8 animals per group were used, 5 females and 3 males for each genotype.

### Human tissue

Formalin-fixed, paraffin-embedded Human AD brains were obtained from the Biobank of the Institute of Medical Genetics and Pathology, Basel University Hospital, Basel, Switzerland.

### Materials

Cell culture media and reagents, if not otherwise stated, were from Gibco (ThermoFisher Scientific, Carlsbad, CA, USA). Plates and flasks were from Nalge Nunc (Rochester, NY, USA). Petri dishes were from Falcon BD (Franklin Lakes, NJ, USA).

4-(4-(2,3-Dihydrobenzo[1,4]dioxin-6-yl)-5-pyridin-2-yl-1H-imidazol-2-yl)benzamide (D4476), 4,5,6,7-Tetrabromo-2-azabenzimidazole, 4,5,6,7-Tetrabromobenzotriazole (TBB) and other chemicals were from Tocris (Bristol, UK) or Merck-Sigma (Darmstadt, Germany).

### Cell culture

The Institutional Animal Care and Use Committee of the San Raffaele Scientific Institute approved the animal use procedures. Primary cultures of hippocampal neurons were prepared according to [3] from 2- to 3-day-old Sprague-Dawley rats. Briefly, after subdivision of hippocampi into small sections, the tissue was incubated into Hank’s solution containing 3.5 mg/ml trypsin type IX (Merck-Sigma) and 0.5 mg/ml DNase type IV (Calbiochem, La Jolla, CA, USA) for 5 min. The pieces were then mechanically dissociated in a Hank’s solution supplemented with 12 mM MgSO_4_ and 0.5 mg/ml DNase IV. After centrifugation, cells were plated onto poly-ornithine-coated coverslips and maintained in minimal essential medium supplemented with 20 mM glucose, B27 (Life Technologies, Carlsbad, CA, USA), 2 mM glutamax, 5% fetal clone III (FCIII; Hyclone, South Logan, UT, USA) and 5 μM 1-β-D-cytosine-arabinofuranoside (Merck-Sigma). Cultures were maintained at 37 °C in a 5% CO_2_ humidified incubator and used between 7 and 15 days after plating.

Primary cultures of cortical astrocytes were established from 1- to 2-day-old Sprague-Dawley rats (Charles River, Wilmington, MA, USA) according to [4]. Briefly, cortices were freshly dissected, cut into small pieces, and washed in Hank’s balanced salt solution supplemented with 10 mM Hepes/Na pH 7.4, 12 mM MgSO_4_, 50 U/ml penicillin, and 50 μg/ml streptomycin (Gibco). Tissue dissociation was performed with trypsin (2.5 mg/ml trypsin type IX, in the presence of 1 mg/ml DNase; 10 min at 37 °C) in two subsequent steps without mechanical trituration and terminated by 1:1 dilution in serum-containing medium. After centrifugation (50 × *g*, 15 min) cells were plated in 75-cm^2^ flasks with Minimum Essential Medium Eagle with Earle’s balanced salt solution, supplemented with 10% donor horse serum, 33 mM glucose, 2 mM glutamax (Gibco), 50 U/ml penicillin and 50 μg/ml streptomycin, and kept at 37 °C in a humidified 5% CO_2_ atmosphere. Two steps of overnight shaking at 230 rpm were performed to induce selective detachment of microglia. After reaching confluence, astrocytes were trypsinized and re-plated onto poly-lysine-coated plastic multiwells. Astrocytes were used within 3 days after re-plating.

Hek293 cells were maintained in Dulbecco’s modified Eagle’s medium supplemented with 10% FCIII, 2 mM glutamine, 10 mM Na pyruvate, 50 U/ml penicillin, and 50 μg/ml streptomycin and kept at 37 °C in a humidified 5% CO_2_ atmosphere. Cells were routinely tested for mycoplasma contamination.

### Expression plasmids

Constructs used for transfections were (i) pcDNA3.1-BACE1 (containing the human BACE1 coding sequence obtained by PCR and cloned EcoRI–XhoI); (ii) pcDNA3.1-5UTR-BACE1 (containing the human BACE1 coding sequence obtained by PCR and cloned EcoRI–XhoI, downstream of the BACE1 5′UTR, cloned KpnI-XhoI from T3pA-B1x—[5]); (iii) pcDNA3.1-5′UTRM2-BACE1 (containing the human BACE1 coding sequence obtained by PCR and cloned EcoRI–XhoI, downstream of the BACE1 5′UTR, cloned KpnI-XhoI from T3pA-B1-mut2—[5]); (iv) pcDNA3.1-Luc+ (containing the firefly luciferase coding sequence cloned XhoI-NotI from pT3a—[5]); (v) pcDNA3.1-5′UTR-Luc+ (containing the firefly luciferase coding sequence cloned XhoI-NotI from pT3a, downstream of the BACE1 5′UTR, cloned KpnI-XhoI from T3pA-B1x—[5]); (vi) pcDNA3.1-5′UTRM2-Luc+ (containing the firefly luciferase coding sequence cloned XhoI-NotI from pT3a, downstream of the BACE1 5′UTR, cloned KpnI-XhoI from T3pA-B1-mut2—[5]); (vii) pcDNA3-Flag4Bwt (kind gift of J. Hershey).

### Cell transfection

HEK293 cells or cortical astrocytes were plated the day before transfection at 50% of confluence in poly-L-Lysine (Sigma-Aldrich) treated 6-well plates and then transfected with 1.8 μg of DNA (1.5 μg of plasmid encoding WT-eIF4B and 300 ng of plasmid encoding GFP) and 4 μl of Lipofectamine 3000 (Life Technologies), according to the manufacturer’s instructions. After 24 h cells were collected, lysed in lysis buffer (phosphate-buffered saline containing 10 mM EDTA/Na, 2% NP-40, 0.2% SDS, CLAP, 1 mM NaF, 2 mM Na_3_VO_4_), total protein content determined with the BCA reagent and then used for luciferase activity detection or directly analysed by western blot.

Primary hippocampal neurons (4 days in vitro) were transfected with 100 pmol of siRNA (ON-TARGETplus NON-TARGETING rat POOL, ON-TARGETplus SMART POOL against rat eIF4B and ON-TARGETplus SMART POOL against rat CK2 alpha - ThermoFisher Scientific) and 4 μl of Lipofectamine 3000 (Life Technologies), according to the manufacturer’s instructions. At 8 days in vitro neurons were collected, lysed in PBS (containing 10 mM EDTA/Na, 1% TX-100, and protease inhibitor cocktail), total protein content determined with the BCA reagent (ThermoFisher Scientific, Pierce), and then analysed by western blotting.

### Luciferase reporter assay

Firefly luciferase (Fluc) and Renilla luciferase (Rluc) activities were revealed with the Dual-Luciferase reporter assay system (Promega) and measured (5 s readings) using a plate-reading luminometer (Mithras, Berthold). The Rluc reporter gene was used to normalize for differences in transfection efficiency.

Fluc reporter gene was used to normalize for differences in transfection efficiency when the vectors containing the BACE1 coding sequence were used. In this case, Fluc activity was revealed with the Single-Luciferase reporter assay system (Promega) and measured (5 s readings) as described above.

### Cellular treatments

When indicated, bicuculline 10 μM (Tocris, Bristol, UK) was added directly in the culture medium, for the specified times. D4476 10 μM and TBB 25 μM were added 2 h before bicuculline or Aβ oligomers treatment, alone or in combination. Aβ42 oligomers were prepared from a human Aβ(1–42) peptide (Bachem, Bubendorf, Switzerland) stock 2.5 mM in DMSO. To prepare oligomers, 2.5 mM Aβ DMSO stock was diluted to 50 μM in EMEM w/o phenol red and left for 24 h at 4 °C. Immediately before the experiment, Aβ oligomers were diluted to 2.5 μM in culture medium and added to the neurons. Control neurons were treated with the same concentration of DMSO in culture medium.

For detection of APP C-terminal fragments cells were treated, as in [6], with 1 μm N-[N-(3,5-difluorophenacetyl)-L-alanyl]-S-phenylglycine t-butyl ester (DAPT) (Merck-Sigma) in DMSO to inhibit gamma-secretase for 15 min before treatment with Bicuculline.

### Western blotting

About 25 μg of proteins were separated by standard SDS-PAGE and transferred onto nitrocellulose membrane. The nitrocellulose filter was stained with Ponceau S (0.2% in 3% trichloroacetic acid) and de-stained with double distilled-water for protein visualization. After 1 h of blocking with TBST (10 mM Tris/HCl, 150 mM NaCl, 0.1% Tween-20) containing 5% bovine serum albumin (Roche Diagnostics, Basel, Switzerland) or skimmed powdered milk, the membranes were incubated overnight with the primary antibodies and, after extensive washing, with horseradish peroxidase-conjugated anti-rabbit or mouse secondary antibody (Bio-Rad, Hercules, CA, USA). For loading controls membranes were stripped in acidic buffer (0.2 M glycine, 0.1% SDS, 1% Tween-20, pH 2.2) and re-probed with the appropriate antibody. In the cases where stripping was not possible, the same lysates were run simultaneously on duplicate gels, and probed with phospho and total antibodies.

Proteins were revealed on auto-radiographic films (GE Healthcare, Piscataway, NJ, USA) or by direct acquisition using the Biorad Chemidoc system by Super Signal West Chemiluminescent Substrate (ThermoFisher Scientific, Waltham, MA, USA). Bands were quantified using ImageJ and protein levels normalized against the loading control. Phosphorylated eIF4B levels were normalized against total eIF4B, then for loading (Tubulin).

The following antibodies were used for western blot: anti-APP (Total APP and 22C11 clone), anti-Flag polyclonal, and anti-alpha-Tubulin antibodies from Merck (Merck KGaA, Darmstadt, Germany); anti-BACE1, ADAM10, eIF4A, eIF4B, CK2 alpha, AKT, phospho Ser473 AKT, p44/42 MAPK (Erk1/2), Phospho-p44/42 MAPK (Erk1/2) (Thr202/Tyr204) from Cell Signaling Technology (Danvers, MA, USA); anti-phospho Ser504 from Abcam (Cambridge, UK). Antibodies against mouse soluble APPα (rat mAb A16M 5G11, specific for murine APPsα in culture media, generated by immunization against DAEF GHDSGFEVRHQKC-COOH peptide—[9]) and β (mouse mAb BAWT antibody specific for APPsβ—[10] were a kind gift of Dr. Stephan Lichtenthaler.

**Table:** 

Antibody	Product Number	Concentration	Blocking	Producer
eIF4B	3592	1:1000	5% BSA in TBST	Cell Signaling Technology
S504-eIF4B	Ab75823	1:3000	5% BSA in TBST	Abcam
BACE	5606	1:1000	5% BSA in TBST	Cell Signaling Technology
Total APP	A8717	1:2500	5% Milk in TBST	Merck
CK2	2656	1:1000	5% BSA in TBST	Cell Signaling Technology
S473-AKT	4060	1:1000	5% BSA in TBST	Cell Signaling Technology
AKT	4691	1:5000	5% BSA in TBST	Cell Signaling Technology
Phospho-ERK	4370	1:1000	5% BSA in TBST	Cell Signaling Technology
ERK	9102	1:1000	5% BSA in TBST	Cell Signaling Technology
alpha Tubulin	T9026	1:6000	5% Milk in TBST	Merck
22C11	MAB348	1:1000	5% Milk in TBST	Merck
eIF4A	2013	1:1000	5% BSA in TBST	Cell Signaling Technology
Adam10	14194	1:1000	5% BSA in TBST	Cell Signaling Technology
Flag	F7425	1:1000	5% Milk in TBST	Merck

### Biochemical procedures

#### Lysis

Cells were lysed by direct addition of 2x SDS-sample buffer (100 mM Tris–HCl, pH 6.8, 5 mM EDTA/Na, 4% SDS, 10% glycerol, 0.4 M DTT, 0.02% bromophenol blue). Slices were collected in PBS and the pellet was lysed with RIPA buffer (150 mM NaCl, 50 mM Tris-Cl (pH 8), 1% Tx-100, 0.5% Na-deoxycholate and 0.1% SDS, protease and phosphatase inhibitor).

#### Tissue homogenization

Brains from 2 months or 6 months mice were homogenized in RIPA buffer with 30 strokes of a glass-Teflon homogenizer and centrifuged at 15,000 × *g*, 4 °C for 15 min. The protein content was analyzed by BCA (ThermoFisher Scientific).

### Organotypic slice culture

Organotypic slice cultures were prepared as described previously [7, 8]. Briefly, young (postnatal days 5–7) C57BL/6J mice were sacrificed by decapitation and brains were isolated. The cerebellum, olfactory bulbs, and mid-brain were removed and the two hemispheres separated and placed onto the cutting stage of the tissue chopper (Mcllwain, model TC752, Mickle Laboratory Engineering Company). 350-μm-thick sagittal sections were cut and further dissected. Intact cortico-hippocampal slices were selected under a dissection microscope (SZ61, Olympus) and incubated for 30 min at 4 °C in pre-cooled dissection media (50% HEPES-buffered MEM, 1% penicillin–streptomycin, 10 mM Tris, pH 7.2). Four slices were then plated onto each 0.4-μm porous polytetrafluoroethylene (PTFE) membrane insert (PICMORG50, Millipore), placed in a 3.5-cm dish with 1 ml of slice culture media containing 50% HEPES-buffered MEM, 25% HBSS, 1 mM L-glutamine (Gibco), and 25% heat-inactivated horse serum (Merck-Sigma) at pH 7.4 and maintained in a cell culture incubator at 37 °C, 5% CO_2_. Media was exchanged 1 day after preparation and subsequently every 3 days. Drugs were applied directly to the slice culture media and reapplied at every media exchange for a total of 7 days of treatment. Slices were treated with 25 μM TBB alone or in combination with 10 μM D4476 dissolved in DMSO or DMSO alone for the controls.

Viability of organotypic slices was assessed via propidium iodide staining and determination of LDH release in the culture medium (CytoTox 96 Non-Radioactive Cytotoxicity Assay, Promega, Madison, Wisconsin, USA).

### Immunohistochemistry

Six-months-old transgenic mice from the APPPS1 and *App*^*NL-G-F*^ lines were anesthetized i.p. with a mixture of ketamine (400 mg/kg) and Xylazine (27 mg/kg) and transcardially perfused with cold 0.1 M PBS for 5 min followed by 4% Paraformaldehyde (PFA) in 0.1 M PBS for 15 min. Brains were then isolated and postfixed in 4% PFA-PBS for 20 min and transferred to 30% sucrose in 0.1 M PBS for cryopreservation at 4 °C. Once the brains sank in the sucrose solution, they were embedded in optimal cutting temperature compound (O.C.T./Tissue-Tek, Sakura), frozen on dry ice, and then kept at −80 °C until sectioning. 30 μm coronal brain sections were cut using a cryostat (CryoSTAR NX70, ThermoScientific) and placed in 0.1 M PBS until staining. Alternatively, sections were kept in anti-freezing solution (30% Glycerol, 30% Ethylenglycol, 10% 0.25 M PO_4_ buffer, pH 7.2–7.4 and 30% dH_2_O) at −20 °C and briefly washed in 0.1 M PBS before staining. Staining was performed in free-floating conditions [11]. Briefly, sections were permeabilized with 0.5% Triton-PBS (PBS-T) for 30 min, then blocked in 5% normal Goat Serum in PBS-T for 1 h and incubated overnight at 4 °C in blocking solution with the corresponding primary antibodies (rabbit anti-mouse eIF4B, 1:100, Cell Signaling Technology; mouse anti-human Aβ-NAB228, 1:500, Santa Cruz; rabbit anti-mouse BACE1, 1:250, Epitomics). After primary antibody incubation, sections were washed three times with PBS-T and incubated with corresponding secondary antibodies (1:500, 555-conjugated goat anti-rabbit and 488-conjugated goat anti-mouse) together with DAPI (1:20:000) to visualize nuclei. Since co-staining with eIF4B and BACE1 antibodies was not possible due to the same species of origin, anti-eIF4B/anti-Aβ-NAB228 and anti-BACE1/anti-Aβ-NAB228 stainings were performed in parallel in contiguous brain sections from the same animals and corroborated in three mice from both mouse models. included as negative controls. Representative ×20 and higher magnification (×63) images were taken using confocal microscopy (z-stack, ×20 dry objective and ×63 water objective, respectively, Leica TCS SP5 II) from similar cortical regions. Z-stack images were converted to maximum intensity projections using the ImageJ software (NIH) for better visualization.

Immunohistochemistry on human formalin-fixed, paraffin-embedded sections was performed using a VENTANA OptiView DAB IHC Detection kit and acquired on a Benchmark Ultra system (Roche Diagnostics, Basel, Switzerland), according to the manufacturer instructions. Consecutive slices were stained with antibodies against human eIF4B, anti-phospho Ser504 eIF4B (Abcam, Cambridge, UK), and anti Amyloid beta (Agilent, Santa Clara, CA, USA). All tissue sections were stained and processed automatically, with the same protocol and in parallel. The concentration of the antibodies and the exposure time were determined as the best possible compromise to reveal eIF4B, phospho Ser504 eIF4B, and amyloid beta contemporaneously in healthy and AD brain sections.

### MEA recordings

Dissociated hippocampal cells were plated on poly-ornithine (400 μg/ml)-treated multielectrode arrays (MEAs; Multi Channel Systems, Reutlingen, Germany) and maintained in the culture medium, previously described, for 10-11 DIV before recordings.

The electrical activity was analyzed at 37 °C using the MEA1060-inv-BC system (Multi Channel Systems MCS, Reutlingen, Germany). Data, recorded at 25 kHz/ch from 60 channels (corresponding to 60 electrodes present in the chip), were filtered from 300 Hz to 3 kHz. The firing rate was analyzed in the active channels (where at least 10 spikes/5 min occurred) and spikes were sorted using a threshold algorithm; this threshold corresponds to multiple of standard deviation of the biological noise during the first 500 ms of the recordings (spike = 5× S.D. noise). The firing rate was evaluated as the number of spikes recorded in a 20 min time window after the administration of 10 μM bicuculline, compared with the number recorded in a 20 min time window before bicuculline treatment.

### Amyloid-β detection

Total Aβ secreted in the culture media was evaluated by the rat-mouse/human Aβ high specific assay kit (Immuno-Biological Laboratories, Tokyo, Japan) according to the manufacturer’s instructions.

### Statistical analysis

Statistical analysis was performed with Prism software version 9.0 (GraphPad Software Inc., La Jolla, CA, USA). Columns in the graphs represent the mean (±SEM). The number of experiments and the *p* value are indicated in the figure legends, biological replicates are shown in graphs as individual points. The normality test (D’Agostino-Pearson) and equal variance test (Brown-Forsythe) were applied. In case of normally distributed data, statistical significance was evaluated (with 95% confidence intervals) by one-way ANOVA followed by Dunnett post hoc test (for multiple comparisons against a single reference group), Newman–Keuls or Tukey’s post hoc test (for multiple comparisons between groups), two-tailed Student *t*-test (for comparisons between two average values); for samples with non-normal distributions, the nonparametric Mann–Whitney U test (for significant differences between two experimental groups) and the Kruskal–Wallis one-way analysis of variance followed by Dunn’s post hoc test (for the analysis of multiple experimental groups) were used. A value of *P*<0.05 was considered to be statistically significant. Sample size was calculated using G Power software (version 3.1), based on effect sizes calculated from our preliminary experiments, with a power of 0.8 and alpha = 0.05.

## Supplementary information

Supplementary Material

Supplementary Figure 1

Supplementary Figure 2

Supplementary Figure 3

Supplementary Figure 4

Supplementary Figure 5

Supplementary Figure 6

Supplementary Figure 7

## Data Availability

All data generated or analysed during this study are included in this published article and its supplementary information files.
