# Supplementary material for: Casein Kinase 2 dependent phosphorylation of eIF4B regulates BACE1 expression in Alzheimer’s disease
Source: Cell Death Dis. 2021 Aug 4;12(8):769. doi: 10.1038/s41419-021-04062-3 (PMC8339060; doi:10.1038/s41419-021-04062-3)
Supplement: Supplementary file 1 — Supplementary Material [file 41419_2021_4062_MOESM1_ESM.docx]

**Figure S1.** (A) Schematic representation of the transfected constructs, containing Firefly luciferase (FLuc) or BACE1 as reporter gene. White boxes represent uORFs and arrows uAUGs. Mutation of the 2^nd^ uAUG to UUG is indicated. (B) Luciferase activity measured in total cell extracts of primary cortical astrocytes, co-transfected with the indicated constructs and a plasmid encoding the renilla luciferase (see materials and methods). The Firefly Luciferase activity was normalized on renilla luciferase activity to account for differences in transfection efficiencies and data represent the mean±SEM of at least three independent experiments, expressed as fold change over control (luciferase). (C) Quantification of BACE1 levels by western blot (inset) in total cell extracts of primary cortical astrocytes, co-transfected with the indicated constructs and a plasmid encoding Firefly luciferase, to normalize for transfection efficiency. Total cell extracts were subjected to luciferase activity measurement and to SDS–PAGE followed by western blot analysis with anti-BACE1 antibody. Blots were then stripped and reincubated with anti-Calnexin antibody as loading control. The intensities of BACE1 band signals were quantified and normalized for loading and for transfection efficiency (on luciferase activity). Results are presented as mean±SEM of at least three independent experiments, with protein levels shown as fold change over control (BACE1). Statistical significance was calculated using Kruskal-Wallis one-way analysis of variance followed by Dunn’s post-hoc test (B, C) (B: n=3 p=0.0145; C: n=3 p=0.0145; * P<0.05;).

**Figure S2.** Effect of eIF4B silencing. Representative western blot with the indicated antibodies of extracts obtained from hippocampal neurons transfected for 96 hours with a control or an eIF4B siRNA pool.

**Figure S3.** Representative western blot and quantification of the indicated proteins in extracts from primary hippocampal neurons transfected with a control or anti-CK2 siRNA pool for 96 h. Protein levels are shown as fold change over control (untreated neurons). Levels of phosphorylated proteins are normalized against the corresponding total protein, then for loading (Tubulin) and shown as fold change over control (untreated neurons). Data are shown as mean ± SEM of at least three independent experiments for each treatment. Statistical significance is calculated using Mann-Whitney U test (B: n=7 p=0.0070; C: n=7 p=0.0012; D: n=6 p=0.0931 ** P<0.01; ***P<0.001).

**Figure S4.** Increased eIF4B expression and phosphorylation in the brains of *App ^NL-G-F^* mice. Representative western blot (A) and quantification (B-F) of the indicated proteins in extracts from 2 (2M) or 6 (6M) months old brains from *App ^NL-G-F^* or Wild type (Wt) mice. Levels of phosphorylated proteins are normalized against the corresponding total protein, then for loading (Tubulin) and shown as fold change over control (untreated neurons). Statistical significance was evaluated by unpaired two-tailed Student’s t-test. (2 months n=6 – 6 months n=8; B: p=0.0003; C: p=0.0348; D: p=0.0007; * P<0.05; ***P<0.001).

**Figure S5.** eIF4B is localized around Aβ plaques in the brains of *App^NL-G-F^* mice. Immunohistochemical analysis of eIF4B expression in the brains of *App^NL-G-F^* mice. 6 months old *App^NL-G-F^* cortex immunostained for eIF4B (red – A, B) or BACE1 (red – C, D) in combination with Aβ (green) revealed an increased localization of eIF4B around plaques, with a distribution resembling the one of BACE1 in dystrophic neurites. Second and fourth column panels are higher magnification images of boxed regions. Scale bars: A-C 150 μm, B-D 50 μm.

**Figure S6.** Ser504-eIF4B is localized around Aβ plaques in the brains of *App^NL-G-F^* mice. Immunohistochemical analysis of eIF4B expression in the brains of *App^NL-G-F^* mice. 6 months old *App^NL-G-F^* hippocampi immunostained for BACE1 (red - A), eIF4B (red – B) or Ser504-eIF4B (S504-eIF4B, red – C) in combination with Aβ (green) revealed an increased localization of phosoho-eIF4B around plaques. Fourth column panels (D) are higher magnification images of boxed regions. Scale bars: A-C 150 μm, D 50 μm.

**Figure S7.** Treatment of organotypic slices with CK inhibitors is not affecting cell viability. LDH release measured in culture media of APPPS1 brain slices treated with CK2 inhibitor (TBB), alone or in combination with CK1 inhibitor (D4476+TBB) or left untreated (NT). Slices were treated for 7 days before analysis.

**References for experimental procedures**

1 Radde, R., Bolmont, T., Kaeser, S.A., Coomaraswamy, J., Lindau, D., Stoltze, L. et al. Aβ42-driven cerebral amyloidosis in transgenic mice reveals early and robust pathology. EMBO Rep **7**, 940–946 (2006)

2 Saito, T., Matsuba, Y., Mihira, N., Takano, J., Nilsson, P., Itohara, S. et al. Single App knock-in mouse models of Alzheimer’s disease. Nat Neurosci **17**, 661–3 (2014)

3 Ryan, T.A. & Smith, S.J. Vesicle pool mobilization during action potential firing at hippocampal synapses. Neuron **14**, 983–989 (1995)

4 McCarthy, K.D. & De Vellis, J. Preparation of separate astroglial and oligodendroglial cell cultures from rat cerebral tissue. J Cell Biol **85**, 890–902 (1980)

5 Mihailovich, M., Thermann, R., Grohovaz, F., Hentze, M.W. & Zacchetti, D. Complex translational regulation of BACE1 involves upstream AUGs and stimulatory elements within the 5′ untranslated region. Nucleic Acids Res **35**, 2975–2985 (2007)

6 Bettegazzi, B., Mihailovich, M., Di Cesare, A., Consonni, A., Macco, R., Pelizzoni, I. et al. β-Secretase activity in rat astrocytes: Translational block of BACE1 and modulation of BACE2 expression. Eur J Neurosci **33**, 236–243 (2011)

7 Daria, A., Colombo, A., Llovera, G., Hampel, H., Willem, M., Liesz, A. et al. Young microglia restore amyloid plaque clearance of aged microglia. EMBO J **36**, 583–603 (2017)

8 Stoppini, L., Buchs, P.A. & Muller, D. A simple method for organotypic cultures of nervous tissue. J Neurosci Methods **37**, 173–182 (1991)

9 Colombo, A., Wang, H., Kuhn, P.H., Page, R., Kremmer, E., Dempsey, P.J. et al. Constitutive α- and β-secretase cleavages of the amyloid precursor protein are partially coupled in neurons, but not in frequently used cell lines. Neurobiol Dis **49**, 137–147 (2013)

10 Kuhn, P.H., Wang, H., Dislich, B., Colombo, A., Zeitschel, U., Ellwart, J.W. et al. ADAM10 is the physiologically relevant, constitutive α-secretase of the amyloid precursor protein in primary neurons. EMBO J **29**, 3020–3032 (2010)

11 Sebastian Monasor, L., Müller, S.A., Colombo, A.V., Tanrioever, G., König, J., Roth, S. et al. Fibrillar Aβ triggers microglial proteome alterations and dysfunction in Alzheimer mouse models. eLife **8**, 9: e54083 (2020)
