# Supplementary figures and images for: Casein Kinase 2 dependent phosphorylation of eIF4B regulates BACE1 expression in Alzheimer’s disease
Source: Cell Death Dis. 2021 Aug 4;12(8):769. doi: 10.1038/s41419-021-04062-3 (PMC8339060; doi:10.1038/s41419-021-04062-3)

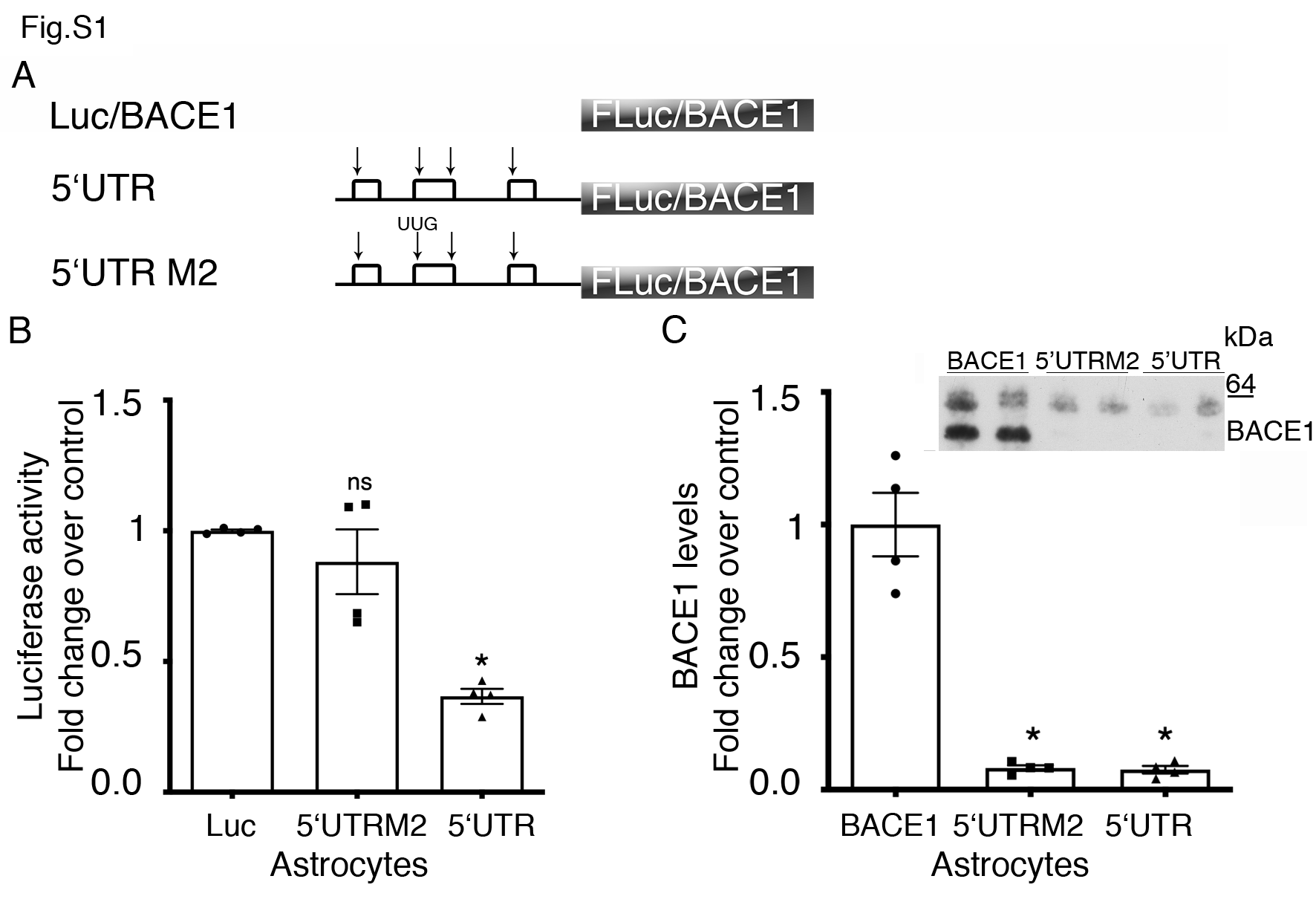

Supplement: Supplementary file 2 — Supplementary Figure 1 [file 41419_2021_4062_MOESM2_ESM.tif]

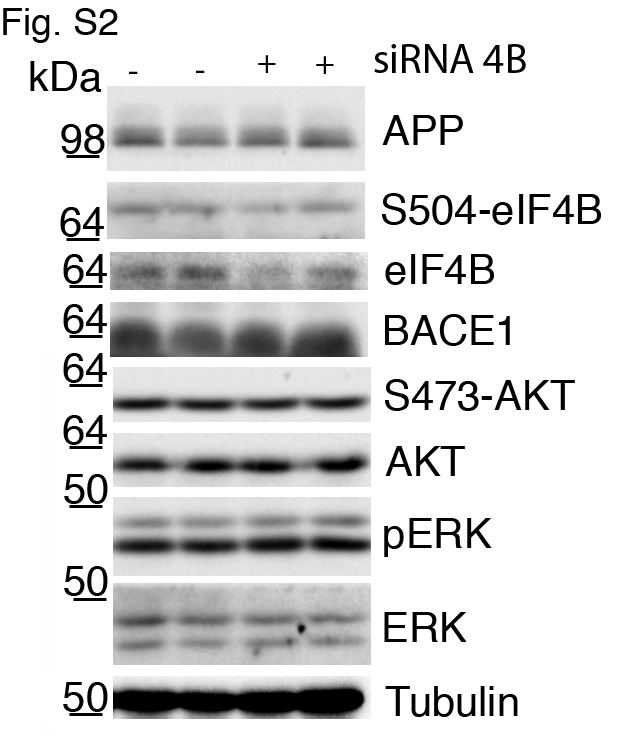

Supplement: Supplementary file 3 — Supplementary Figure 2 [file 41419_2021_4062_MOESM3_ESM.tif]

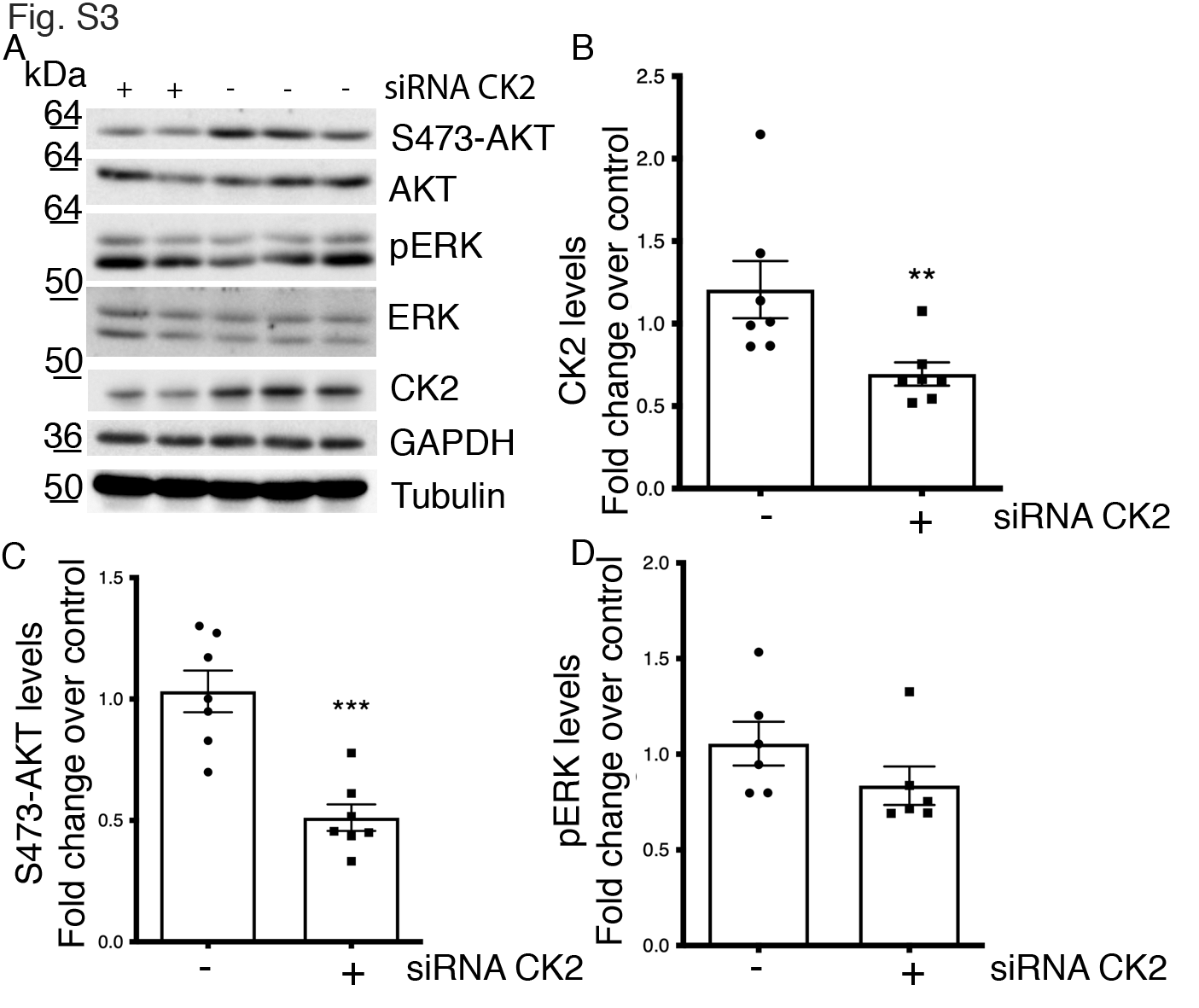

Supplement: Supplementary file 4 — Supplementary Figure 3 [file 41419_2021_4062_MOESM4_ESM.tif]

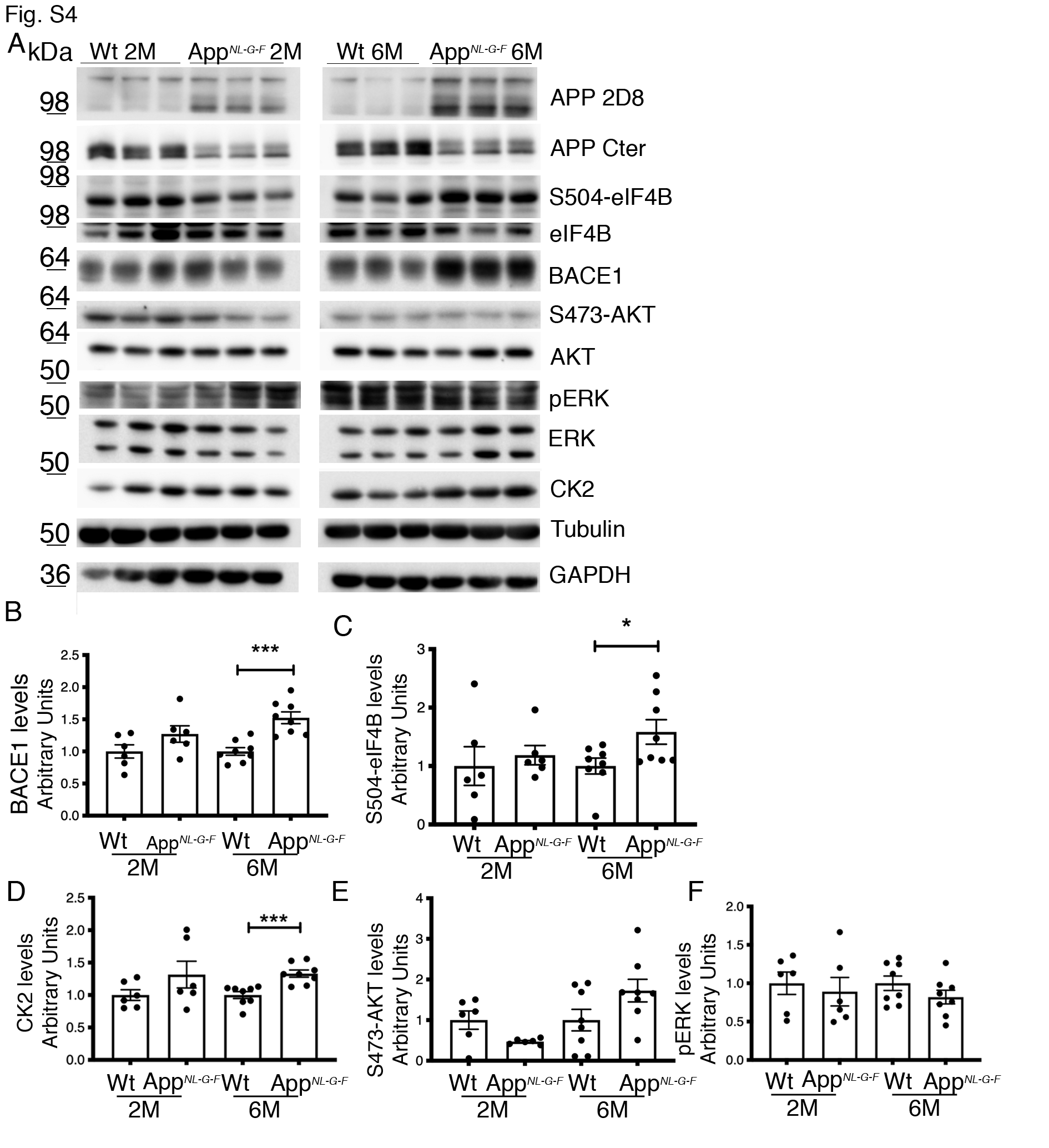

Supplement: Supplementary file 5 — Supplementary Figure 4 [file 41419_2021_4062_MOESM5_ESM.tif]

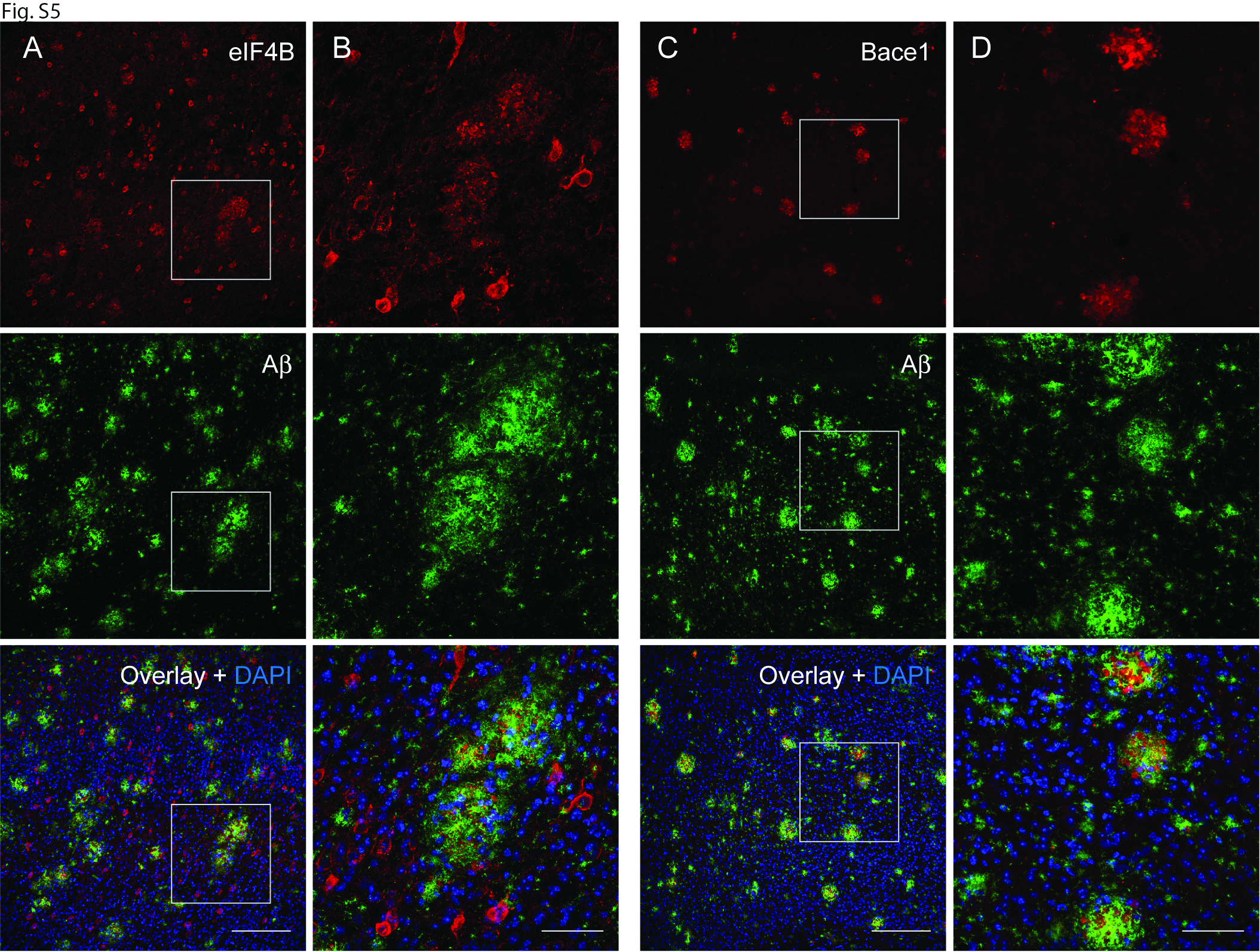

Supplement: Supplementary file 6 — Supplementary Figure 5 [file 41419_2021_4062_MOESM6_ESM.tif]

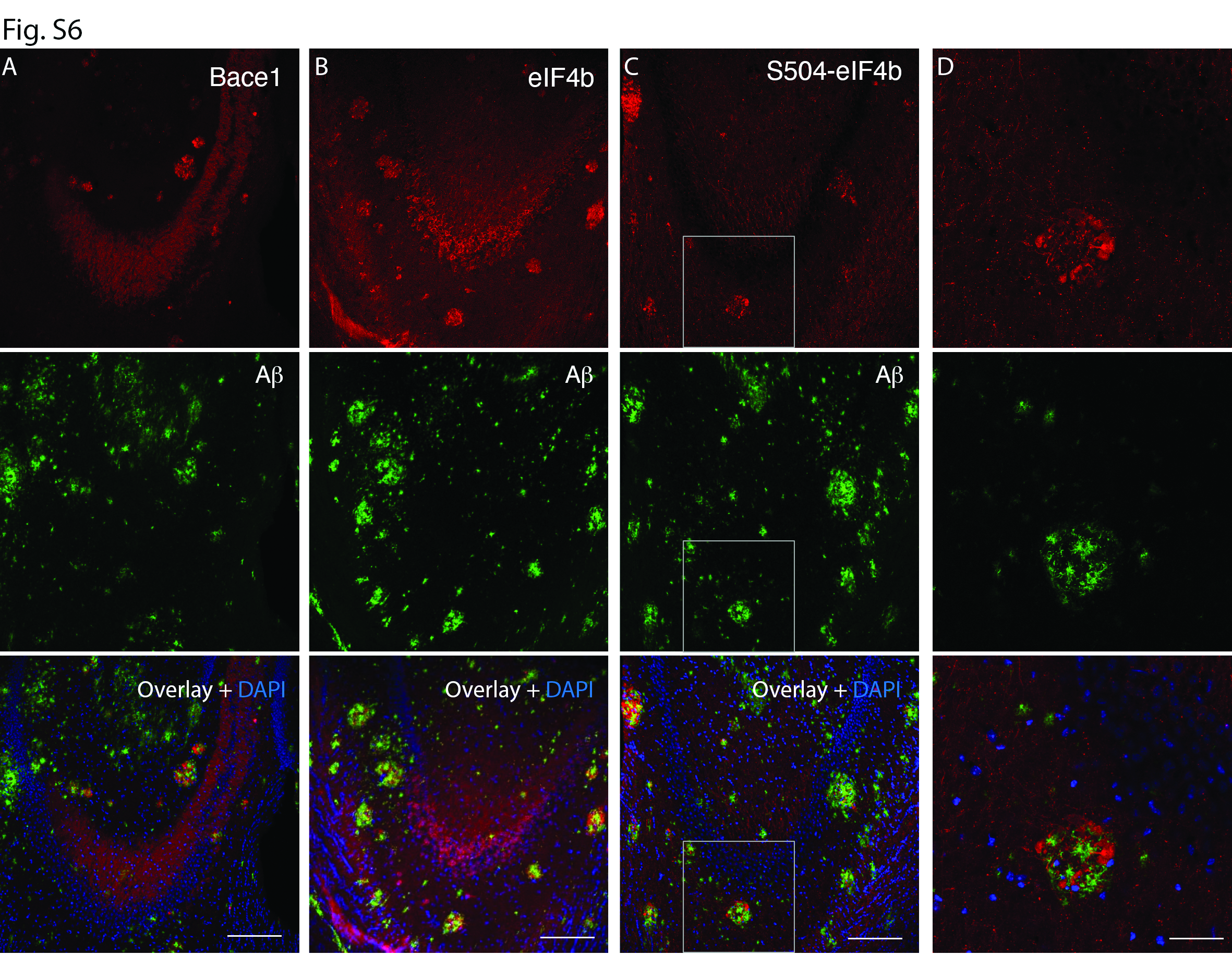

Supplement: Supplementary file 7 — Supplementary Figure 6 [file 41419_2021_4062_MOESM7_ESM.tif]

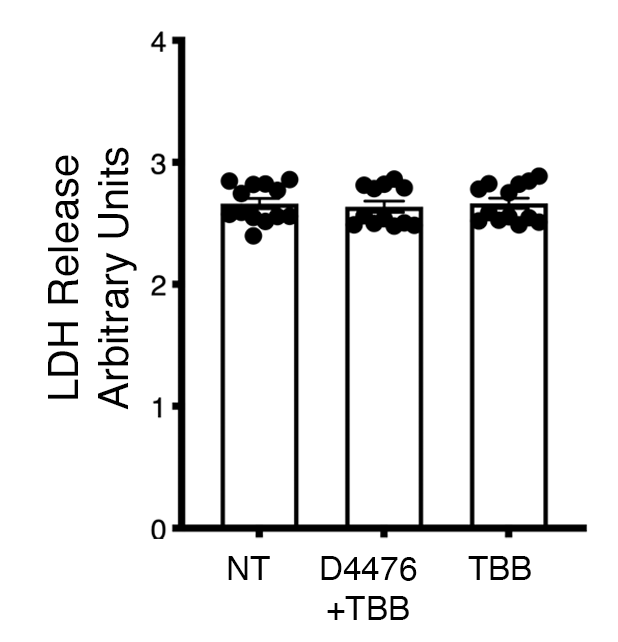

Supplement: Supplementary file 8 — Supplementary Figure 7 [file 41419_2021_4062_MOESM8_ESM.tif]
